# Bulk and Spatially Resolved Extracellular Metabolome of Free-Living Nitrogen Fixation

**DOI:** 10.1128/aem.00505-22

**Published:** 2022-06-02

**Authors:** Darian N. Smercina, Young-Mo Kim, Mary S. Lipton, Dusan Velickovic, Kirsten S. Hofmockel

**Affiliations:** a Biological Sciences Division, Earth and Biological Sciences Directorate, Pacific Northwest National Laboratory, Richland, Washington, USA; b Environmental Molecular Sciences Laboratory, Pacific Northwest National Laboratory, Richland, Washington, USA; c Department of Agronomy, Iowa State University, Ames, Iowa, USA; Georgia Institute of Technology

**Keywords:** soil microbial ecology, multiscale, spatially resolved, nitrogen fixation, microbial metabolomics, GC-MS, MALDI MSI, MALDI, metabolomics, microbial ecology, soil microbiology

## Abstract

Soil nitrogen (N) transformations constrain terrestrial net primary productivity and are driven by the activity of soil microorganisms. Free-living N fixation (FLNF) is an important soil N transformation and key N input to terrestrial systems, but the forms of N contributed to soil by FLNF are poorly understood. To address this knowledge gap, a focus on microorganisms and microbial scale processes is needed that links N-fixing bacteria and their contributed N sources to FLNF process rates. However, studying the activity of soil microorganisms *in situ* poses inherent challenges, including differences in sampling scale between microorganism and process rates, which can be addressed with culture-based studies and an emphasis on microbial-scale measurements. Culture conditions can differ significantly from soil conditions, so it also important that such studies include multiple culture conditions like liquid and solid media as proxies for soil environments like soil pore water and soil aggregate surfaces. Here we characterized extracellular N-containing metabolites produced by two common, diazotrophic soil bacteria in liquid and solid media, with or without N, across two sampling scales (bulk via GC-MS and spatially resolved via MALDI mass spec imaging). We found extracellular production of inorganic and organic N during FLNF, indicating terrestrial N contributions from FLNF occur in multiple forms not only as ammonium as previously thought. Extracellular metabolite profiles differed between liquid and solid media supporting previous work indicating environmental structure influences microbial function. Metabolite profiles also differed between sampling scales underscoring the need to quantify microbial scale conditions to accurately interpret microbial function.

**IMPORTANCE** Free-living nitrogen-fixing bacteria contribute significantly to terrestrial nitrogen availability; however, the forms of nitrogen contributed by this process are poorly understood. This is in part because of inherent challenges to studying soil microorganisms *in situ*, such as vast differences in scale between microorganism and ecosystem and complexities of the soil system (e.g., opacity, chemical complexity). Thus, upscaling important ecosystem processes driven by soil microorganisms, like free-living nitrogen fixation, requires microbial-scale measurements in controlled systems. Our work generated bulk and spatially resolved measurements of nitrogen released during free-living nitrogen fixation under two contrasting growth conditions analogous to soil pores and aggregates. This work allowed us to determine that diverse forms of nitrogen are likely contributed to terrestrial systems by free-living nitrogen bacteria. We also demonstrated that microbial habitat (e.g., liquid versus solid media) alters microbial activity and that measurement of microbial activity is altered by sampling scale (e.g., bulk versus spatially resolved) highlighting the critical importance of quantifying microbial-scale processes to upscaling of ecosystem function.

## INTRODUCTION

Nitrogen (N) is often the most limiting nutrient in soil systems and as such constrains terrestrial net primary productivity with impacts ranging from agricultural production to climate change mitigation (1–3). Soil N availability is governed by N transformations mediated by soil microorganisms whose metabolic activity impacts scales across orders of magnitude, driving energy and nutrient transfer between the atmosphere, biosphere, and pedosphere (4–6). For example, free-living nitrogen fixation (FLNF), the biological conversion of atmospheric N to biologically available forms by heterotrophic soil bacteria, is a key microbially driven process in the terrestrial N cycle with inputs at the microbial scale that influence N availability at the ecosystem scale (7, 8). FLNF represents an important N source for many terrestrial systems (8) with recent estimates suggesting FLNF contributes over one-third of all N fixed via biological nitrogen fixation (BNF) globally (9).

FLNF is carried out by a wide diversity of soil bacteria and occurs in all terrestrial biomes (7, 8). N inputs from FLNF are generally thought to occur through extracellular release of ammonium as is observed with symbiotic N fixation (10–13). However, FLNF occurs under very different environmental conditions than symbiotic N fixation and these conditions are likely to influence the forms of N from diazotrophic, or N-fixing, cells (7). For example, FLNF is thought to occur predominately in the rhizosphere where carbon (C) is readily available, but has also been measured in bulk soil (7, 8) where it may be occurring in saturated pores or on the surfaces of soil aggregates. Each of these soil microhabitats will have unique environmental conditions such as availability of C and oxygen to which FLNF activity is sensitive (7) and are therefore likely to influence N inputs from FLNF. Much remains unknown about FLNF, including the forms of N released to the environment during FLNF and the influence of environmental conditions on these contributions.

**FIG 1:**
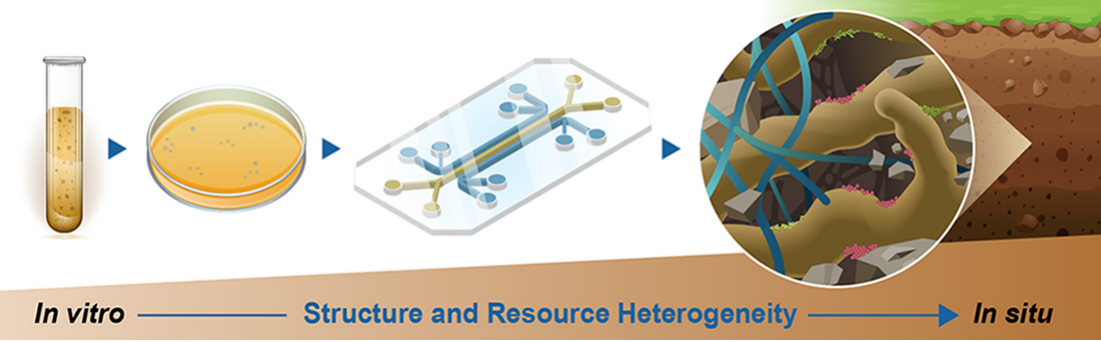
Depiction of the systematic scaling of system complexity from liquid and solid culture to synthetic soils (represented here as a microfluidic chip) to *in situ* soil conditions. In our study, we focus on the first two steps, relating metabolomics in liquid and solid media culture.

Establishing the relationship between diazotrophic bacteria, their release of N-containing metabolites, and the feedbacks on bulk N processes remains a key challenge in soil microbial ecology. Despite generating large amounts of data through routine application of multiomic techniques, quantitatively linking omics of a specific function (e.g., functional genes and proteins), like FLNF, to process rate measures of that function is often unsuccessful (5, 6, 14–16). This is likely because of the inherent challenges of studying soils and soil microorganisms (15–18). For example, sampling of multiomic data in soils often yields material not only from active and functioning microbial community members, but also dead/inactive members and extracellular material (e.g., relic DNA and proteins) in the environment making it difficult to distinguish which community members are actively carrying out a function (6, 16, 17). Additionally, the vast differences in temporal and spatial scales between the microbial scale and the sampling scale used to measure process rates, like FLNF, make understanding mechanisms governing microbial activity and quantifying the impact on ecosystem functions difficult (18). Thus, quantitatively linking soil microorganisms and their activity to the ecosystem functions they perform requires systematic and hierarchical characterization of soil microorganisms and their functions across scales of space and complexity (Fig. 1) (14, 19).

*In vitro* studies using pure cultures or limited species are an appealing option for addressing this challenge and have the potential to provide fundamental microbial and ecological knowledge (20–22). However, culturing conditions can be quite different from those experienced by microorganisms in soil. Liquid culture, for example, lacks available structure for microbial attachment to surfaces, which has been shown to impact microbial growth and function (23, 24), but may be similar to saturated pore environments in soil. Similarly, solid culture can provide attachment surfaces and a more heterogenous environment than liquid culture, thus may be similar to soil aggregate surfaces. Overall, the presence or absence of physical structure influences microbial function, and it is therefore essential to systematically characterize function *in vitro* under different growth conditions. By comparing growth conditions of increasing structural complexity with parallels in the soil environment, we aim to determine how culture work may better inform *in situ* processes (Fig. 1).

In this study, we explore three questions: (i) What forms of N do diazotrophic bacteria contribute to their environment? (ii) Do these N forms collectively create a metabolic signature unique to FLNF function? (iii) How do growth conditions and sampling scale influence the forms of N contributed by FLNF and the presence of metabolic signatures of FLNF? We examined extracellular N-containing metabolites from diazotrophic bacteria cultured individually under conditions that promote (N-free; no added N) or inhibit (N-rich; ~1.33 g N L^−1^ as tryptone) FLNF. Tryptone, a complex source of amino acids, was used as the N source in this study to represent organic N, the most abundant form of N in soils (25, 26). Unlike inorganic N sources, organic N can also act as a C source, but in our study we aimed to overcome reliance of bacteria on C from tryptone by providing ample C in a media specifically designed to support the high C demands of diazotrophic cultures (27). Two diazotrophic bacteria with distinct growth strategies (e.g., Gram-negative versus Gram-positive) common in soils were chosen for this study, Azotobacter vinelandii and Paenibacillus polymyxa.

To examine the impact of growth conditions on detected metabolites, cultures were grown in liquid media, representing saturated pore spaces in soil, or on solid media, representing soil aggregate surfaces. A homogenous environment, such as that of saturated pores, may provide better access to C and support greater FLNF rates compared to a more heterogenous environment, like soil aggregate surfaces. FLNF rates are in turn likely to drive the form of N contributions to soil systems where greater FLNF rates may result in more direct release of N-containing metabolites like ammonium as observed with symbiotic BNF (10–12), while lower FLNF rates may result in N contributions through biomass turnover (28). Additionally, to understand the impact of sampling scale, we measured extracellular metabolites from solid media cultures at two sampling scales: spatially resolved measures (μm scale) using matrix-assisted laser desorption/ionization mass spectrometry imaging (MALDI–MSI) and bulk sampling (~cm scale) via gas chromatography–mass spectrometry (GC-MS) analysis. We hypothesized: (i) N-containing extracellular metabolites from N-free treatments would indicate FLNF contributes N as ammonium and a variety of organic N compounds, (ii) N-containing metabolic profiles would be distinct between N-free and N-rich conditions demonstrating unique metabolic signatures associated with FLNF activity, (iii) distinct N-containing metabolite profiles would be detected between liquid and solid culture conditions, (iv) the same N-containing metabolites would be detected at bulk and spatially resolved scales.

## RESULTS

### Microbial biomass: total biomass, biomass C, and biomass N.

**FIG 2:**
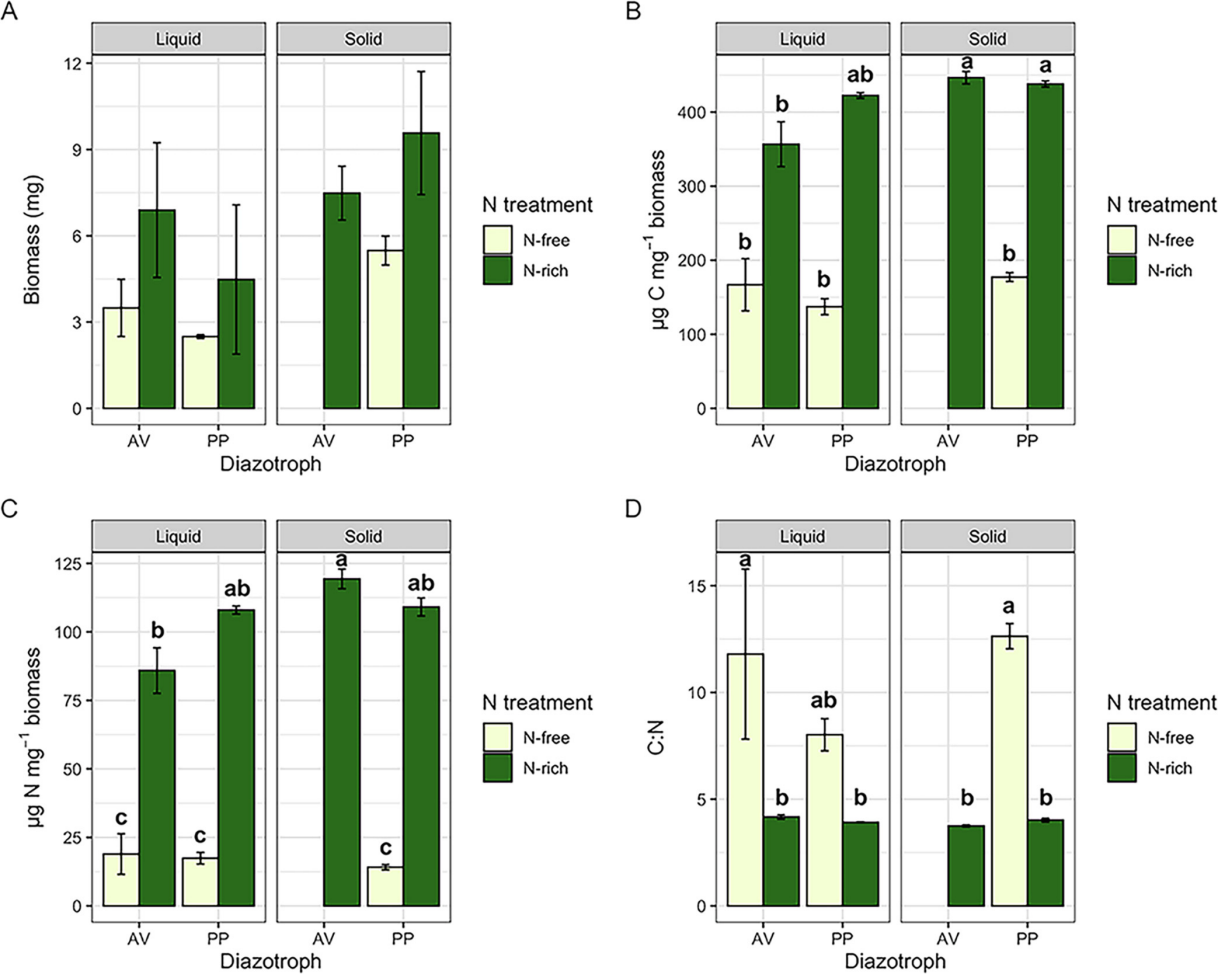
Microbial (A) biomass, (B) C content, (C) N content, and (D) C:N ratio of A. vinelandii (AV) and *P. polymyxa* (PP). Bars represent average values ± standard error and are colored by nitrogen treatment. Figures are faceted by culture type. Lowercase letters represent significant difference at *P* < 0.05. Note that results for the AV, N-free solid treatment are not presented because biomass could not be collected.

Total microbial biomass, including cells and associated debris such as exopolysaccharides (EPS), was collected from a total of 24 samples (2 organisms × 2 N treatments × 2 media types × 3 replicates, plus cell extracts) to account for any biomass related differences in observed extracellular metabolite pools. Total microbial biomass was measured in all samples except those in the A. vinelandii N-rich solid treatment where microbial colonies had grown into and below the agar surface and it was not possible to collect biomass. Total biomass was highly variable across all treatments (coefficient of variation across treatments ranged from 4.6% to 100.4%) and there were no significant differences observed with N treatment (N-free or N-rich), culture type (liquid versus solid media), or organism (A. vinelandii or *P. polymyxa*) (Fig. 2A). Both biomass C (Fig. 2B) and biomass N (Fig. 2C) tended to be greater in N-rich treatments compared with N-free treatments. Interestingly, the abundances of C and N in N-free biomass were notably low which may indicate greater abundances of heavier elements in the biomass such as iron, molybdenum, phosphorus, and oxygen resulting from physiological differences in N-fixing cells, including accumulation of elements like iron and molybdenum to support nitrogenase production (29), greater abundance of intracellular adenosine triphosphate (ATP) (30), or alteration of membrane phospholipids (31, 32). There was also a trend toward greater biomass C and N from solid media, but this was mostly observed in the N-rich treatments. These C and N values translated to C:N ratios that predominately differed only between N treatments with the N-free treatment resulting in greater C:N ratios of biomass than N-rich conditions, regardless of culture type or organism (Fig. 2D).

### Extracellular ammonium availability.

**FIG 3:**
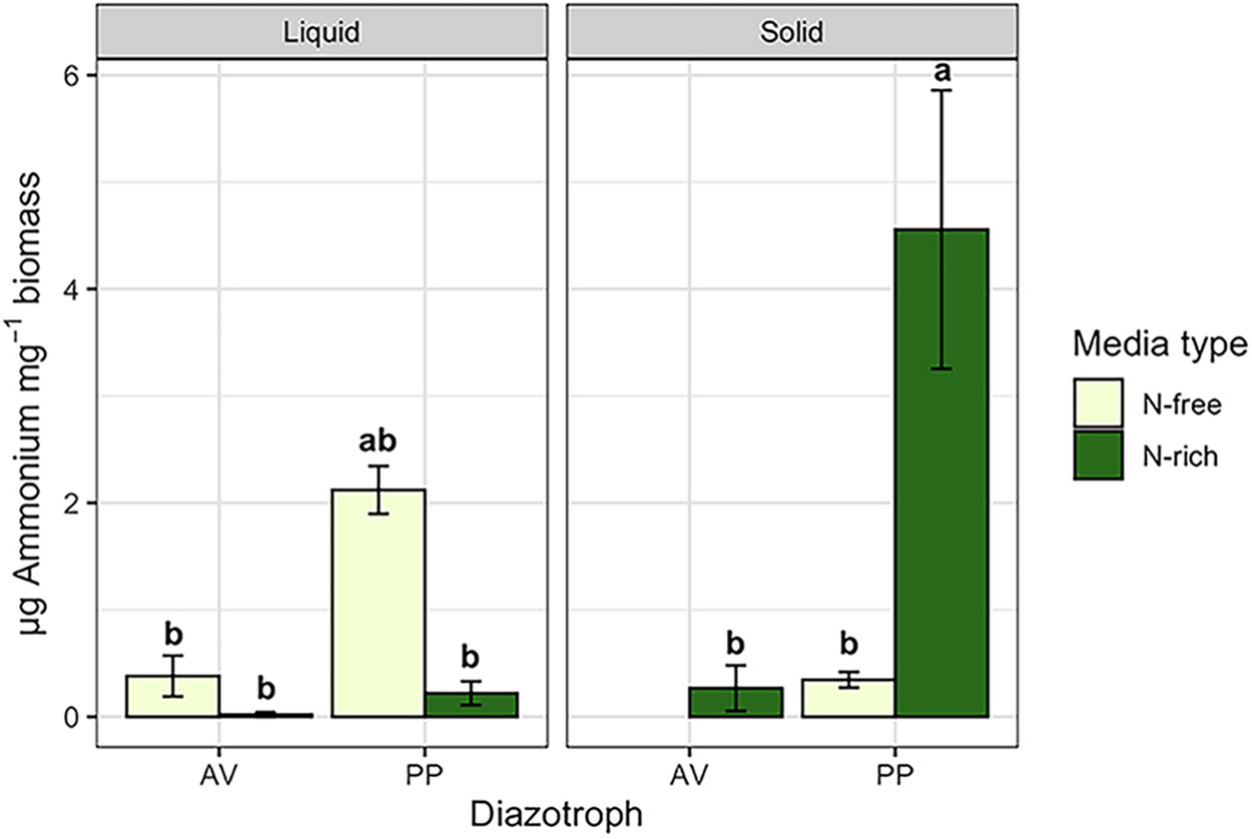
Extracellular ammonium availability per mg microbial biomass of A. vinelandii (AV) and *P. polymyxa* (PP). Bars represent average values ± standard error and are colored by nitrogen treatment. The figure is faceted by culture type. Lowercase letters indicate significant difference at *P* < 0.05. Note that results for the AV, N-free solid treatment are not presented because biomass could not be collected.

**FIG 4:**
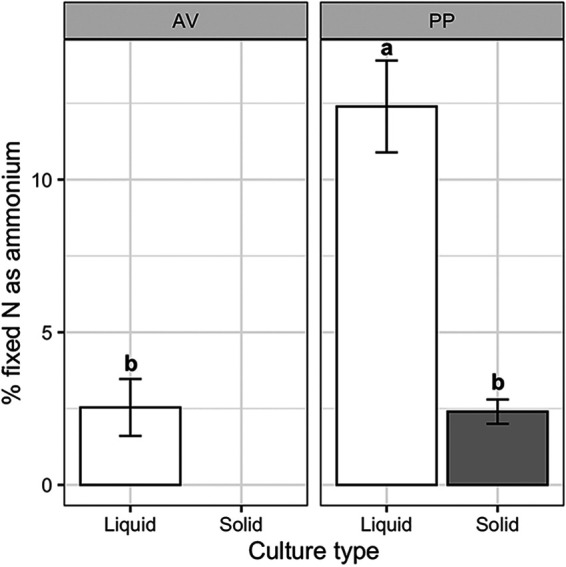
Percent of fixed nitrogen available as extracellular ammonium in N-free treatments calculated at μg ammonium/μg biomass N * 100 Bars represent average ± standard error and are colored by culture type. Figures are faceted by organism, A. vinelandii (AV) and *P. polymyxa* (PP). Lowercase letters indicate significant differences at *P* < 0.05. Note that results for the AV solid treatment are not presented because biomass could not be collected.

Extracellular ammonium availability was measured in supernatant and rinsate samples and was detected in all treatments regardless of culture type, N treatment, or organism. On a per unit biomass basis, ammonium concentrations were significantly greater in *P. polymyxa* compared with A. vinelandii (*F* = 16.390, *P* = 0.0012) with significant culture type and N treatment interactions (*F *= 35.411, *P* < 0.0001). Extracellular ammonium availability per unit biomass was overall eight times greater in *P. polymyxa* than A. vinelandii cultures (Fig. 3). Under N-free conditions, ammonium availability in *P. polymyxa* cultures tended to be greater in liquid than in solid culture while in N-rich conditions the opposite was observed (Fig. 3). No significant difference was observed by N treatment or culture type for A. vinelandii (Fig. 3). We also calculated the percentage of fixed N available as extracellular ammonium as μg extracellular ammonium per μg fixed N, where fixed N is estimated as total biomass N for N-free treatment samples (Fig. 4). We found 2.39% to 12.40% of fixed N is readily available as extracellular ammonium depending on organisms and culture conditions where *P. polymyxa* in liquid culture was greater than all other treatments.

### Bulk extracellular metabolites.

Across all treatments (N, culture type, and organism), 307 metabolites were detected with bulk sampling via MPLEx extraction and of these 93 were successfully annotated (>80% confidence). The total number of detected metabolites differed between treatment groups (Fig. S1) with generally more metabolites detected in N-free treatments, the majority of which were within the unannotated portion of detected metabolites. Because FLNF activity is hypothesized to result in the release of N-containing metabolites, we focused on N-containing extracellular metabolites. Of the 93 annotated metabolites detected through bulk sampling, 35 were identified as N-containing.

**FIG 5:**
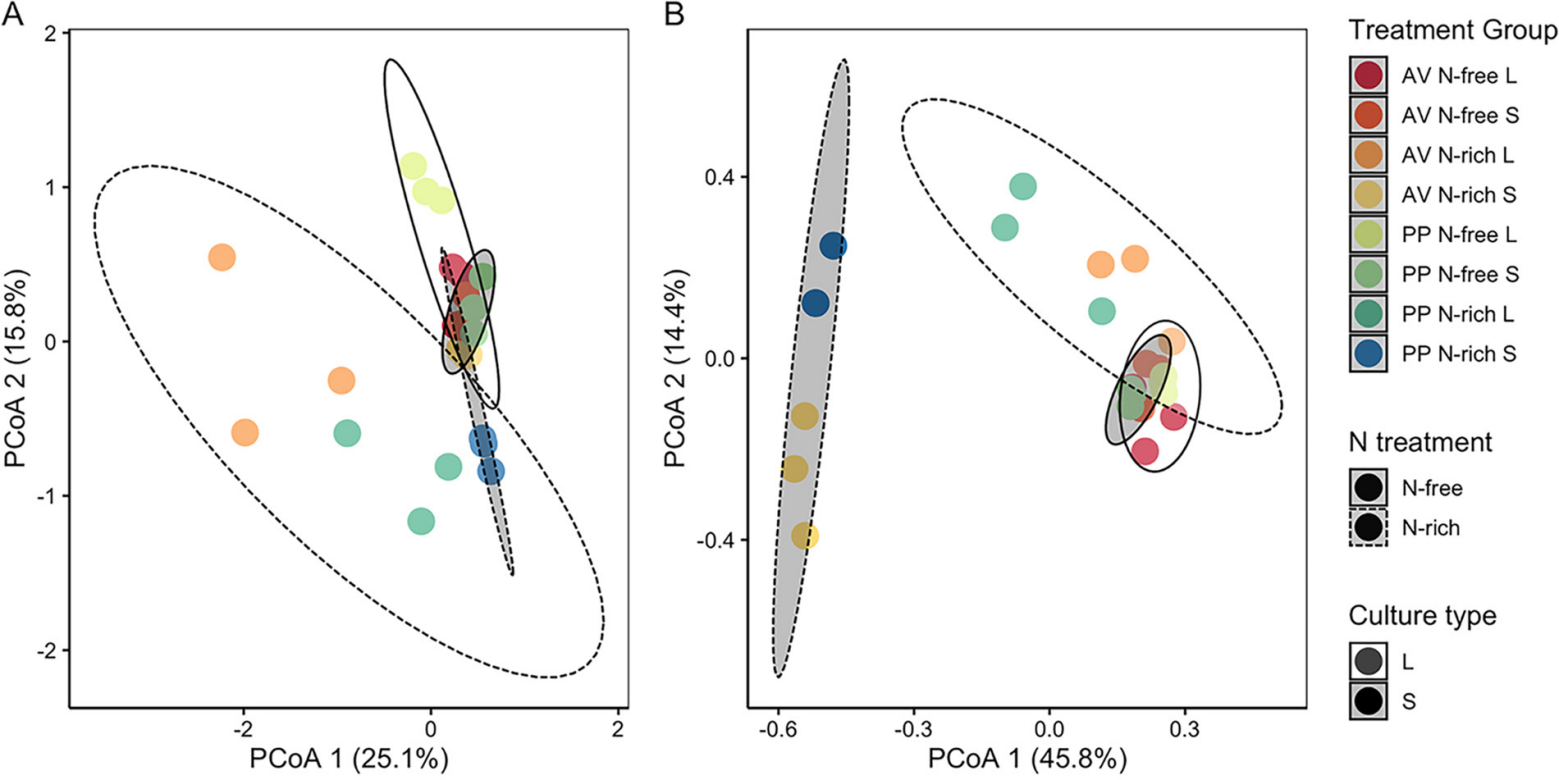
Principal coordinates analysis (PCoA) of metabolite chemistry based on (A) Euclidean distance of peak intensity and (B) Jaccard distance of presence-absence including all identified N-containing metabolites from all samples. Each point represents a single sample and are colored by treatment group (organism [A. vinelandii, AV or P. polymyxa, PP]; N treatment [N-free or N-rich]; culture type [liquid or solid]). 95% confidence ellipses are shown for culture type, represented by color, and N treatment, represented by line type.

**FIG 6:**
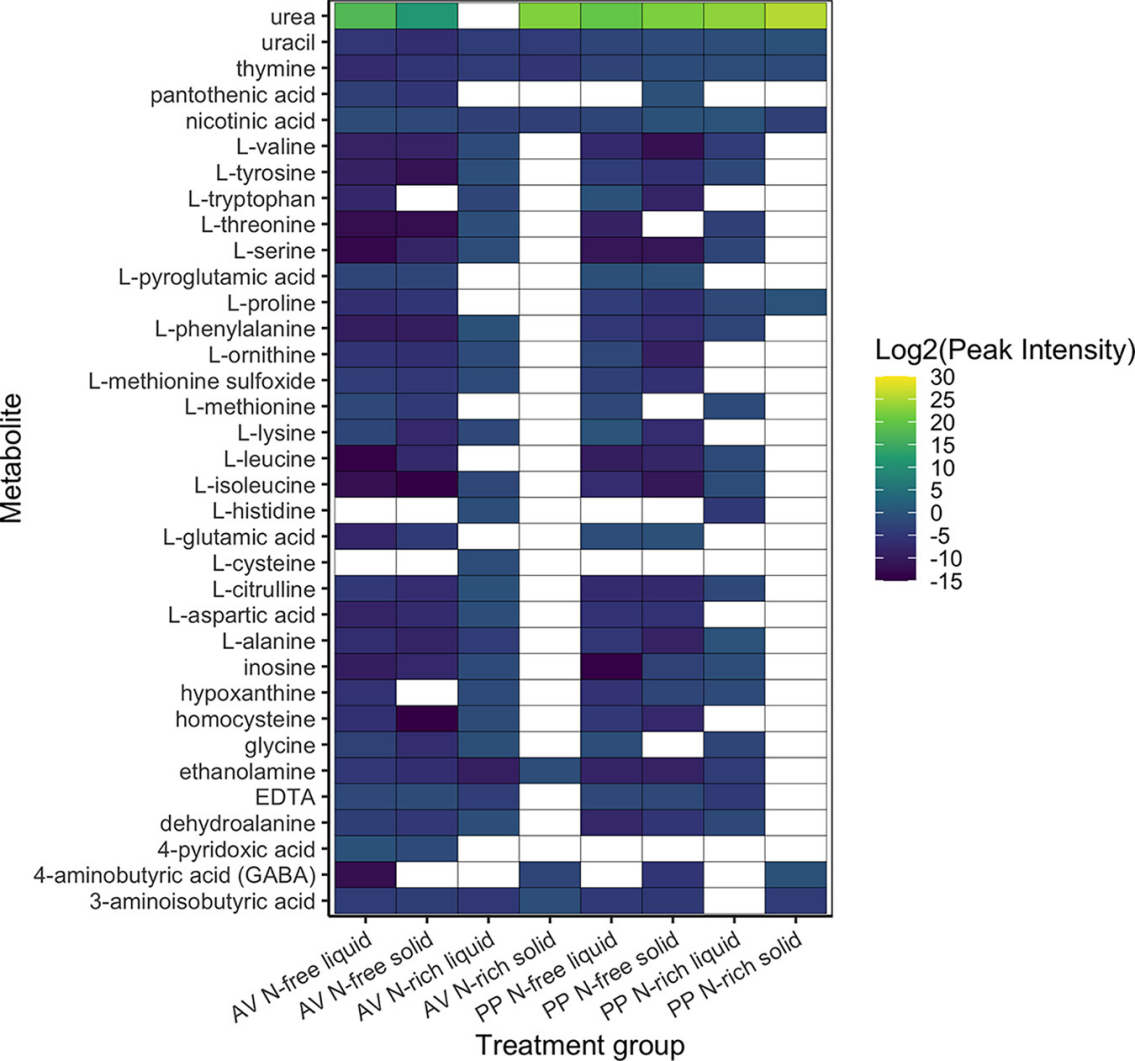
Heatmap of N-containing metabolites and their peak intensities across treatment groups (organism [A. vinelandii, AV or *P. polymyxa*, PP]; N treatment [N-free or N-rich]; culture type [liquid or solid]). White cells indicate metabolite peak intensities was below detection. Peak intensity values are shown as log_2_ transformed to improve visualization. Additionally, all biological replicates are presented in Fig. S6.

Distinct metabolite profiles, represented by Euclidean and Jaccard distance based on all identified N-containing metabolites, were observed between N treatment, culture type, and their interaction (Fig. 5; Table 1). Metabolite profiles based on peak intensities separated predominantly by N treatment and then by culture type, but with strong overlap in the N-free treatment for each culture type (Fig. 5A). Metabolite profiles based on presence-absence show clear separation between culture types for N-rich treatments but have little separation under N-free conditions (Fig. 5B). Overall, N-free treatments of both organism and culture type tended to be richer in N-containing metabolites than N-rich treatments (Fig. 6; Fig. S2) but had similar or significantly lower total peak intensities of N-containing metabolites compared to N-rich treatments (Fig. S3). Examining the specific composition of these N-containing compounds, we found a variety of amino acids in N-free samples not well represented in N-rich samples (Fig. 6), but only a few N-containing metabolites were unique to N-free conditions, including pantothenic acid, L-pyroglutamic acid, l-glutamic acid, and 4-pyridoxic acid.

**TABLE 1:** PERMANOVA results for Euclidean and Jaccard distance of macroscale peak intensities and presences of N-containing metabolites

Effect	*df*	Sums of squares	Mean squares	F model	R squared	*P*–value
Euclidean						
N treatment	1	7.203	7.204	4.169	0.134	0.0001
Culture type	1	6.617	6.617	3.829	0.123	0.0001
Interaction	1	5.258	5.258	3.043	0.098	0.0001
Residual	20	34.559	1.728		0.644	
Total	23	53.638			1	
Jaccard						
N treatment	1	1.458	1.458	13.504	0.278	0.0001
Culture type	1	0.861	0.861	7.968	0.164	0.0001
Interaction	1	0.773	0.773	7.161	0.147	0.0002
Residual	20	2.160	0.108		0.411	
Total	23	5.252			1	

### Spatially resolved extracellular metabolites.

**FIG 7:**
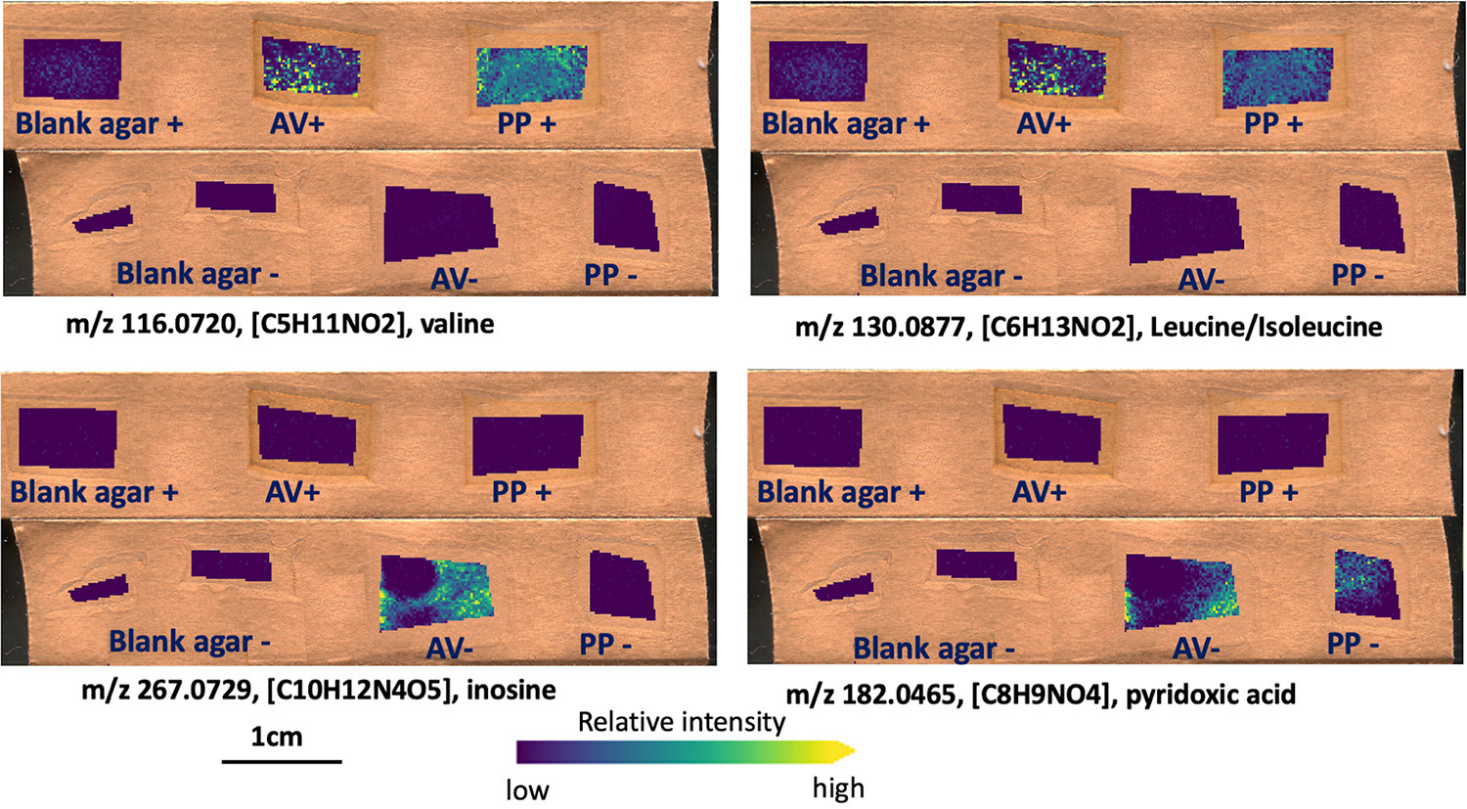
Examples of the N-containing metabolites detected at the microscale using MALDI MSI. Each measured region represents an organism*N-treatment on solid media (organism [A. vinelandii, AV or *P. polymyxa*, PP]; N treatment [N-free. – or N-rich, +]). This also includes cell-free media samples (Blank + = N-rich media; Blank – = N-rich media). All ions are annotated as [M–H]- adducts. Ion images of individual *m/z* values were generated on the same color bar scale for visual comparison in terms of relative ion abundance.

Across all treatments, METASPACE analysis identified 69 metabolites in spatially resolved samples analyzed via MALDI MSI of which 41 were N-containing. Compared with metabolites detected at the bulk scale, only a few potential amino acids were detected at this resolved microbial scale, including l-leucine and l-valine and these were only at detectable concentrations within the N-rich treatment (Fig. 7) unlike the diverse array of amino acids detected in bulk samples in association with N-free treatments. Observationally, N-free treatments seemed to be characterized by unique presence of organic acids rather than N-containing compounds. However, we did identify a few N-containing compounds unique to N-free treatments at the microbial scale, including inosine and 4-pydroxic acid (Fig. 7). Inosine was detected in N-free treatments of both A. vinelandii and *P. polymyxa* and was not at detectable levels in N-rich samples. Also, much like bulk sampling scale detection, 4-pyridoxic acid was exclusively detected in A. vinelandii N-free treatment samples.

## DISCUSSION

We explored the impact of N availability and growth conditions on the extracellular metabolome of diazotrophic bacteria between two sampling scales (bulk versus spatially resolved sampling). We found evidence of extracellular organic and inorganic N contributions from FLNF demonstrating that terrestrial N contributions from FLNF are likely to occur in multiple forms. In general, we found growth conditions and FLNF activity alter extracellular metabolite profiles and influence the detection of metabolites at bulk and spatially resolved scales.

### Nitrogen contributions from FLNF.

Products of BNF by symbiotic diazotrophs are well-studied and typically observed as ammonia and ammonium with contested evidence for production of amino acids (10–13). This knowledge of symbiotic BNF is thought to translate directly to FLNF leading to the assumption that free-living diazotrophs also excrete ammonia/ammonium into the surrounding environment during BNF. However, intracellular ammonia produced during FLNF is rapidly assimilated through conversion to glutamine or glutamate via the glutamine synthetase (GS) and glutamate synthetase (GOGAT) pathways (33). Thus, excreted ammonium would necessarily be in excess of these assimilation pathways (33). Ammonium excretion has been observed in wild-type Azotobacter vinelandii DJ, at concentrations between ~2 and ~25 μM (34, 35), values within range of those measured in this study (Fig. S4). However, in many cases, measurable ammonium excretion was only observed from Azotobacter vinelandii cultures genetically altered to disrupt the GS-GOGAT pathways or facilitate constitutive nitrogenase synthesis (30, 36–38).

An alternative hypothesis to ammonium excretion is that N contributions occur as organic N, either through direct release of N-rich compounds like amino acids (10, 13) or through turnover of dead biomass (28). Our bulk metabolomics data support this hypothesis with many N-containing organic compounds, including amino acids detected in N-free treatments. In fact, N-free treatments tended to be richer in N-containing compounds than N-rich treatments, particularly when comparing against N-rich solid media which had few N-containing metabolites (Fig. S2). The structure of our study did not allow us to determine whether these organic molecules were directly excreted by active, N-fixing cells or released during cell turnover. However, other metabolites detected in the system suggest cell turnover contributed at least partially to this N release. For example, we detected inosine in both bulk and spatially resolved analysis, and it was unique to N-free treatments in spatially resolved samples. Inosine, a metabolic product of adenine degradation likely indicates salvage activities by the bacterial populations (39, 40) and could indicate freely available nucleotides from cell lysis and turnover. FLNF may therefore contribute available N through increasing microbial biomass and turnover, but this needs to be verified in future studies. Regardless of whether these N-containing compounds are actively excreted or released after cell death, this metabolic exchange with the surrounding environment indicates that terrestrial N contributions from FLNF are more diverse and complex than previously thought.

### Identification of metabolic signatures.

Through bulk and spatially resolved analysis, we found few N-containing metabolites exclusive to N-free treatments. At the bulk scale, these include pantothenic acid, L-pyroglutamic acid, l-glutamic acid, and 4-pyridoxic acid. We similarly find 4-pyridoxic acid at the spatially resolved microbial scale as well as nine other metabolites, including inosine. 4-pyridoxic acid was unique to A. vinelandii N-free treatments at the microbial scale. However, despite being uniquely associated with N-free treatments and therefore microbial populations actively fixing N, it may be difficult to assign these as a signature of FLNF function. Of these compounds, only l-glutamic acid has a direct association with the FLNF pathway. Other metabolites seem more indicative of microbial nutrient needs and function. For example, pantothenic acid, vitamin B_5_, is involved in the synthesis of coenzyme A and is a coenzyme for many reactions involved in protein and lipid metabolism (41–43). This is particularly important for the processing of organic acids like malate, the main C source provided in this study. Thus, the detection of vitamin B_5_ is likely indicative of malate metabolism via the TCA cycle and its unique detection in the N-free treatment suggests a higher respiration rate in these N-fixing populations than in the N-rich populations. Increased respiration is a common response among diazotrophs in oxygenated environments as a protection mechanism to prevent or reduce denaturation of nitrogenase via oxygen (7, 44, 45). We also identified 4-pyridoxic acid, a derivative of pyridoxine (vitamin B_6_). Pyridoxine is a key cofactor in amino acid, fatty acid, and carbohydrate metabolisms, but can also act an oxygen protectant (43). During this redox reaction, pyridoxine degrades and can result in 4-pyridoxic acid. A. vinelandii has been observed to produce B vitamins while under diazotrophic conditions and this seems to be a hallmark of FLNF for this organism (43, 46, 47). Though not directly associated with the N-fixation pathway, these vitamins may tangentially indicate bacteria functions surrounding FLNF such as oxygen regulation and highlight the need to analyze bacterial function holistically rather than focusing on single reactions or pathways.

Additionally, the limited number of unique extracellular metabolites detected in N-free treatments suggests some microbial functions may not have detectable or unique signatures, in the form of extracellular metabolites. This is an important consideration when applying metabolomics to the study of complex soil systems. Soil metabolomics are increasingly being used to study soil microbial ecology and biogeochemical function and have been successfully applied to soil C cycling (48–51). However, metabolites are by definition the by-products of and substrates for metabolic function, and turnover rapidly in soils (52, 53). Therefore, typical soil extractions to collect extracellular components (e.g., K_2_SO_4_ extracts, leachate) (54, 55) only capture what is not consumed by the microbial community. This includes metabolites available in dissolved organic matter pools at the time of sampling and metabolites readily exchangeable from mineral surfaces (56). In both cases, metabolites could be temporally separated from their originating processes making it difficult to trace back the associated metabolic pathway. It could be even more challenging to capture metabolic signatures from nutrient-limited communities, such as those in bulk soil. Under nutrient-limited conditions, resulting metabolic products are likely to be rapidly assimilated or, in the case of processes like FLNF, not released to the surrounding environment. The potential signature compounds of FLNF found here (e.g., amino acids and B-vitamins) are also not uniquely produced by FLNF processes and would be difficult to directly link to FLNF *in situ*. The authors acknowledge our study system may provide a biased view on this issue, being a closed incubation system unlike soils where metabolites may diffuse away from microbes and persist in the environment. However, these findings highlight a key need to understand the soil microhabitat (19).

Lastly, our work highlights the importance of considering growth conditions and their association to *in situ* conditions as we observed clear differences in metabolite profiles between liquid culture, representative of saturated soil pores and solid culture, representative of soil aggregate surfaces. Interestingly, though culture type strongly influenced metabolite profiles, it played a secondary role to N treatment in influencing the number of detected metabolites. This was particularly notable when N was readily available, where presence-absence based profiles were distinct between liquid and solid culture, but only under N-rich conditions. These differences in metabolite profiles were likely not driven by differences in biomass production as culture type had small and nonsignificant impacts on microbial metrics, like total biomass, and biomass C and N content. Thus, these responses seem specifically associated with the presence or absence of physical structure in the environment. Additionally, these findings suggest nutrient limitation, as experienced in the N-free treatments, may be a stronger driver of microbial activity than physical structure and simplified liquid culture may be informative to nutrient-limited *in situ* conditions.

### Implications for upscaling from microbial scale to bulk sampling.

The combination of techniques used in this study allowed us to explore the detection of metabolites across scales from spatially resolved, relevant to microorganisms, to bulk, relevant for soil microbial ecology analysis. MALDI MSI allowed us to resolve the presence of extracellular metabolites on solid media at a microbial scale. Using GC-MS, we were able to evaluate detection of extracellular metabolites at the bulk scale. While the detection ranges of these two techniques do not fully overlap (50 to 500 *m/z* for GC-MS and 92 to 700 *m/z* for MALDI), many metabolites of interest to this study are measurable with both techniques providing valuable information about metabolite detection and sampling scale. It is also important to note that a lack of detection is not equivalent to metabolite absence but only indicates metabolite concentrations were below detection.

Through bulk sampling, we found a wide variety of N-containing compounds in N-free samples, but generally lower peak intensities of N-containing compounds than in the N-rich treatment. While N-containing compounds are characteristic of N-free samples at a bulk scale, these treatments had fewer N-containing metabolites when spatially resolved at the microbial scale. Interestingly, there was a shift in amino acid detection between spatially resolved and bulk scales where amino acids were commonly detected in N-free samples at bulk scale, but in N-rich samples when spatially resolved. This somewhat counterintuitive result highlights differences in N competition at the microbial scale and its influence on bulk measurements.

First, detection of a diverse array of amino acids in the N-free treatment in the bulk sample, but not in the spatially resolved samples, suggests N competition at the microbial scale resulted in rapid uptake of amino acids, while extraction of the bulk metabolite pool likely captured the cumulative low abundance signal of the entire system. Amino acids are shown to have short residence times in soils and experience rapid uptake and turnover (57, 58). In the case of microbial versus bulk scale, it is likely the spatially resolved pool of extracellular amino acids collected from microbial colonies (~200 μm spatial resolution) was small and often below detection. However, in bulk sampling of millions of cells, a larger pool of amino acids coupled to our sampling method could have allowed amino acids to diffuse away and accumulate to detectable levels.

Second, biofilm formation is likely to influence diffusion of metabolites into the surrounding environment (59, 60). Bacteria tend to live in biofilms in their natural environments rather than as individually dispersed cells (61). However, the impact of surrounding environmental conditions, including nutrient availability, on biofilm production is unclear. For example, some studies suggest nutrient-limiting conditions may promote greater biofilm formation (62), while others suggest biofilm formation is greater under more favorable growth conditions (63, 64). This is particularly notable for diazotrophs as biofilms can play a role in oxygen protection (44); thus, investment in biofilm could be beneficial to FLNF activity. Yet, under severe N limitation imposed by an N-free environment the high energy demands of FLNF may limit investment in biofilm. While not directly measured in this study, we noted solid agar plates of Azotobacter vinelandii and Paenibacillus polymyxa cultures had visually greater biofilm formation under N-rich than N-free conditions. Thus, diffusion of amino acids away from populations would have been more easily achieved in the N-free treatment. This is evidenced by the similarity between metabolite chemistry in liquid and solid culture under N-free treatments (Fig. 5B). Similarly, a small number of amino acids were detected in spatially resolved samples from N-rich treatments, but not in bulk samples for the similar N-rich solid media treatment. This could have resulted from greater biofilm formation under N-rich conditions and limited diffusion of small molecules away from cell populations.

The detection of small molecules across sampling scales has important implications for the influence of soil microbial communities on their surrounding environment. In general, our results indicate microbial-scale processes drive bulk metabolite availability. The N-rich treatment in this experiment is an optimal environment and most representative of C and nutrient-rich soil environments like the rhizosphere or detritusphere. Our findings suggest these conditions would result in production of valuable small molecules, like N-rich amino acids, potentially exchangeable with the immediate environment, but biofilm formation may limit diffusion far into the soil environment. Under limiting conditions of the N-free treatment, similar those of bulk soil, microbial activity produces valuable metabolites, like amino acids, but competition between microbes reduces the exchange of these molecules. Understanding how these differences in microbial-scale conditions influence microbial activity and detectability of function is crucial to accurately linking microbe and ecosystem.

### Conclusions.

We demonstrated extracellular production of inorganic and organic N during FLNF and reveal the importance of habitat conditions and sampling scale when quantifying microbial activity. Across bulk and spatially resolved sampling scales, we found FLNF activity to result in N contributions from extracellular ammonium and a variety of organic N compounds. We also identified N-containing metabolites uniquely associated with FLNF activity, including several B vitamins, which may play roles in mitigating oxygen damage to nitrogenase. Despite finding unique metabolites and potential metabolic signatures, many detected metabolites are not exclusively produced through FLNF related pathways, thus would be difficult to assign to FLNF for *in situ* soil samples. This would likely hold true for other processes under nutrient-limited conditions where metabolic products are rapidly assimilated and not captured during sampling. Our findings highlight the need to carefully consider both environmental conditions and sampling scale when quantifying microbial function. We found culture conditions to be a key driver of metabolite chemistry under N-rich and N-free conditions, indicating presence or absence of physical structure in the environment influences microbial processes. Across scales, our results indicate high N competition at the microbial scale under N-free conditions, while at the bulk scale N appeared readily available within the microbial environment. These differences in environmental conditions across sampling scales could lead to incorrect interpretations of microbial function as immediate conditions surrounding microorganisms will drive their activity and may not necessarily match what is measured through bulk or composite sampling.

## MATERIALS AND METHODS

### Culture conditions.

Two diazotrophic bacteria, Azotobacter vinelandii (ATCC BAA 1303) and Paenibacillus polymyxa (ATCC 842), were cultured in this study. Both organisms are commonly found in soils and their genomes are fully sequenced (65, 66). Bacteria were cultured under N-free (no added N) and N-rich (+tryptone) conditions, respectively, promoting or inhibiting FLNF. Nfb media, commonly used to isolate diazotrophs (27), was used for N-free treatments, and was supplemented with tryptone for N-rich treatments. Tryptone was chosen as an N source representative of organic N, an important N source in soils, including in both the rhizosphere and bulk soil (26). Both treatments contained 1.79 g C L^−1^ as malic acid. N-rich media contained tryptone which added ~1.33 g N L^−1^ and ~4.4 g L^−1^ of additional C. Malic acid is a common C sources used to isolate diazotrophic bacteria and the C source typical of Nfb media (27). Cultures were grown in liquid or solid agar media, both representing 5 mL of media. All media was autoclave sterilized prior to inoculation.

Thirty samples were cultured (2 organisms × 2 N treatments × 2 media types × 3 replicates, plus cell extracts) for bulk analysis with an additional set of 14 solid media samples (2 organisms × 2 N treatments × 3 replicates, plus cell extracts) for spatially resolved analysis. Cultures were grown in a temperature-controlled incubator at 25°C to 10^7^ CFU mL^−1^, based on liquid cultures OD600, and then harvested for analysis of extracellular metabolites at two scales—bulk sampling via MPLEx extraction and GC-MS (67) and spatially resolved sampling via colony analysis with MALDI MSI. Extracellular ammonium availability and microbial biomass, including total microbial biomass, biomass C and biomass N, were also measured. Biomass C and biomass N values were used to create biomass C:N ratios. Because FLNF activity is necessary for microbial growth under N-free conditions, measures of total biomass and biomass N are used as estimates of FLNF (27).

### Sample collection.

Extracellular metabolites were collected from liquid culture by centrifuging culture tubes to pellet cells and collecting the resulting supernatant for bulk analysis as described below. Cell pellets were resuspended in autoclave sterilized nanopure water, immediately flash frozen on liquid nitrogen, and stored at −80°C until further analysis. Extracellular metabolites were collected from solid media for bulk and spatially resolved analysis. Bulk samples were collected by washing culture plate surfaces with autoclave sterilized nanopure water and collecting the resulting rinsate. Samples were collected for spatially resolved analysis as described below. Lastly, microbial colonies from rinsate plates were collected from the surface by gentle scraping, transferred to autoclave sterilized nanopure water, flash frozen on liquid nitrogen, and stored at −80°C until further processing.

### Microbial biomass: total biomass, biomass C, and biomass N.

Frozen cell pellets and colonies were lyophilized until completely dry and weighed to obtain total biomass, including cells and associated debris such as EPS. Dried biomass was ground using sterile steel beads and then analyzed for C and N content on a VarioTOC Cube (Elementar, Langenselbold, Germany).

### Extracellular ammonium availability.

We measured extracellular ammonium concentrations in supernatant and rinsate samples using a high-throughput colorimetric ammonium assay (68). Briefly, samples were pipetted in triplicate into clear 96-well plates and incubated with ammonium salicylate and ammonium cyanurate reagents to facilitate color change via the Berthelot reaction. Plates were read for absorbance at 610 nm on a Synergy H1 plate reader (BioTek Instruments, Inc., Winooski, VT, USA).

### Bulk metabolomics–GC-MS.

Bulk metabolomics analysis was conducted on 1-mL subsamples of undiluted, supernatant, and rinsate samples. Supernatant and rinsate samples were prepared for metabolite analysis via GC-MS following the MPLEx protocol for simultaneous metabolite, protein, and lipid extraction (67). Additionally, 1 mL of supernatant and rinsate from sterile liquid culture and solid culture plates were also extracted via MPLEx as cell extracts to account for any metabolites present in the background. This extraction method allows simultaneously collection of metabolites, lipids, and proteins; however, lipid fractions were not analyzed in this study. Additionally, protein yields were too low for downstream analysis. Metabolite samples were completely dried under speed-vacuum concentrator and chemically derivatized prior to analysis by GC-MS as reported previously (69). The *m/z* range of derivatized metabolites scanned was 50 to 550 *m/z* which can detect organic acids, amino acids, and mono- to trisaccharides. Raw GC-MS data were processed using the PNNL in-house metabolomics database, which can identify metabolites using two-dimensional matching factors (fragmented spectrum + retention index) (70), and with cross-checking against commercially available NIST 20/Wiley 11th GC-MS spectral databases (67, 71).

### Spatially resolved metabolomics–MALDI MSI.

Samples were prepared for spatially resolved analysis via MALDI-MSI using a previously described workflow (72). Briefly, areas of agar were excised from Petri dishes and placed onto double-sided adhesive copper tape adhered to indium tin oxide (ITO)-coated glass slides (Bruker Daltonics; Fig. S5). Total sampled areas ranged from 6.8 to 51.5 mm^2^ at 200 μm resolution (Table S1). This approach enhanced our sensitivity for analysis in negative ionization mode and improved adhesion of agar onto the MALDI target. Samples were dried at room temperature overnight, then treated with MALDI matrix using a HTX TM-Sprayer (HTX Technologies). For analysis in negative-ion mode, 7 mg mL^−1^ of N-(1-naphthyl) ethylenediamine dihydrochloride (NEDC) in 70% MeOH was sprayed with eight passes at 1,200 μL min^−1^, 75°C, a spray spacing of 3 mm, and a spray velocity of 1,200 mm min^−1^. MALDI-MSI was performed on a 15-Tesla Fourier transform ion cyclotron resonance (FTICR)-MS (Bruker Daltonics, Billerica, MA, USA) equipped with SmartBeam II laser source (355 nm) using 200 shots pixel^−1^ with a frequency of 2 kHz and a step size of 200 μm. FTICR-MS was operated to collect *m/z* 92 to 700, using a 209-ms transient, which translated to a mass resolution of *R* ~70,000 at 400 *m/z*. Metabolites in this range can typically be detected to fmol concentrations.

### Data analysis.

A factorial ANOVA with N treatment, culture type, organism, and their interactions as main effects followed by a Tukey’s *post hoc* test was used to determine treatment differences for measured variables. Prior to statistical analysis, bulk metabolite values were blank corrected by subtracting peak intensities identified in cell extracts of the associated treatment. Differences in bulk N-containing metabolite profiles were evaluated using distance matrices based on range scaled, peak intensities (Euclidean) and presence-absence (Jaccard) generated from all detected metabolites in all samples using R *vegan* (73). For Euclidean distance, peak intensities were represented as zero when a metabolite abundance was below detection. Differences between culture type, N treatment, and organism were determined via PERMANOVA using *adonis* in R *vegan*. Spatially resolved metabolite data were acquired using FlexImaging (v 4.1, Bruker Daltonics), and image processing, segmentation, colocalization analysis, and visualization were performed using SCiLS (Bruker Daltonics). The list of *m/z* values that colocalized with the colonies were uploaded to the METLIN (https://metlin.scripps.edu) for putative molecular annotations based only on accurate *m/z*, secured by using a 3-ppm window during the search. imzML files (created by SCiLS) of our analyses were also uploaded to METASPACE (74) for metabolite annotation based on both accurate *m/z* and a comprehensive bioinformatics framework that considers the relative intensities and spatial colocalization of isotopic peaks as well as quantifies spatial information with a measure of spatial chaos followed by the estimation of the false discovery rate. For this purpose, we used KEGG-v1 and NPA-2019-08 (Natural Product Atlas) databases that are available in METASPACE. METASPACE uses by default 3-ppm window in its annotation engine.

### Data availability.

The GC-MS data sets generated for this study can be found in the Open Science Framework (OSF) depository at https://osf.io/fmy7g/. MALDI MSI detected metabolites have been uploaded to METASPACE and can be accessed under data set 20210803_dv_smerchina_neg.
