# Supplementary material for: Bulk and Spatially Resolved Extracellular Metabolome of Free-Living Nitrogen Fixation
Source: Appl Environ Microbiol. 2022 Jun 2;88(12):e00505-22. doi: 10.1128/aem.00505-22 (PMC9238392; doi:10.1128/aem.00505-22)
Supplement: Supplemental file 1 — Fig. S1 to S6 and Table S1. Download aem.00505-22-s0001.pdf, PDF file, 1.2 MB [file aem.00505-22-s0001.pdf]

Supplemental Material

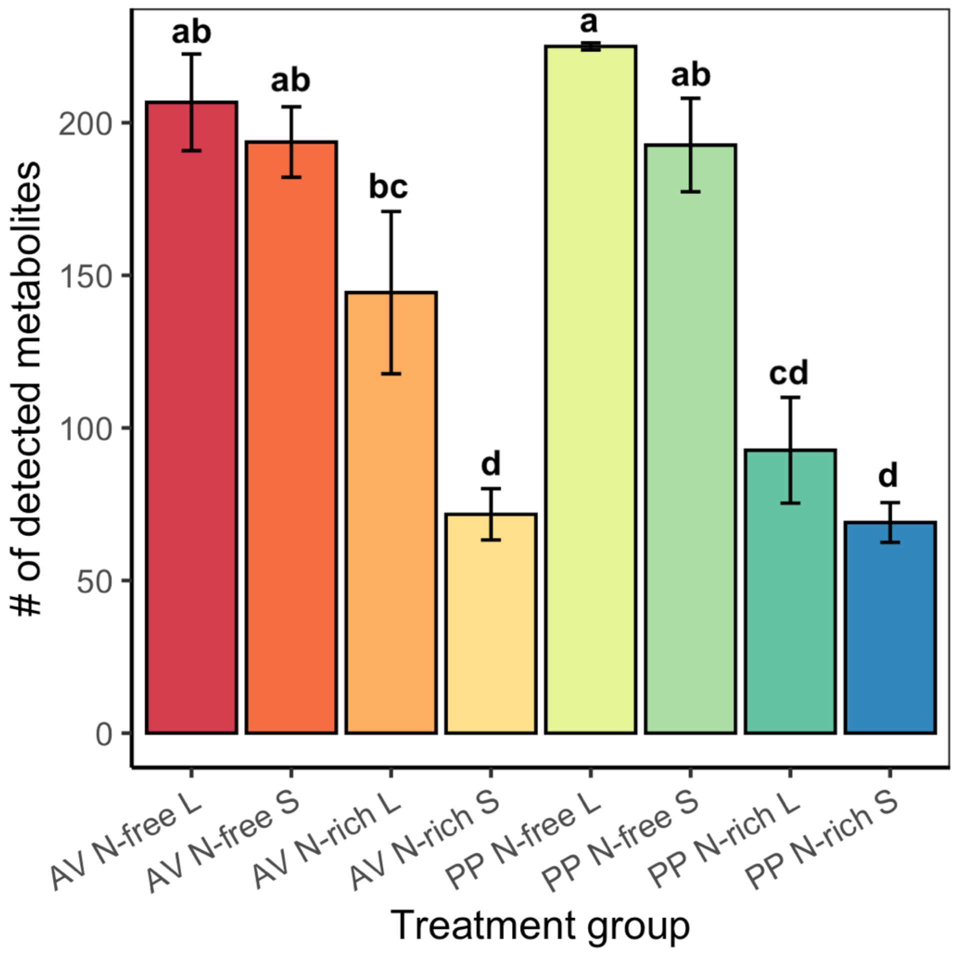

Fig. S1: Number of detected metabolites at the bulk macroscale across treatments. Bars represent average ± standard error. Lowercase letters indicate significant difference at $p < 0.05$.

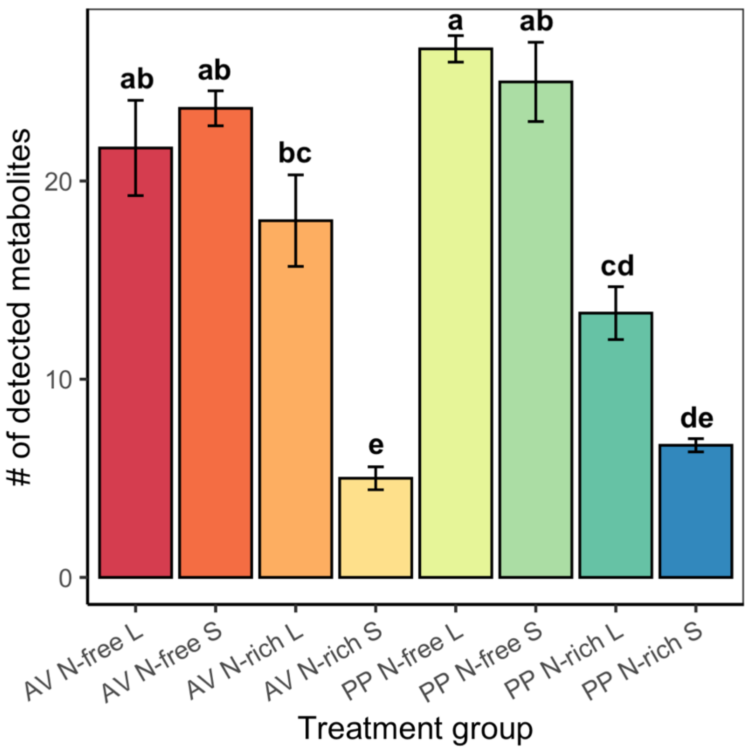

Fig. S2: Number of identified N-containing metabolites at the bulk macroscale across treatments. Bars represent average ± standard error. Lowercase letters indicate significant difference at $p < 0.05$.

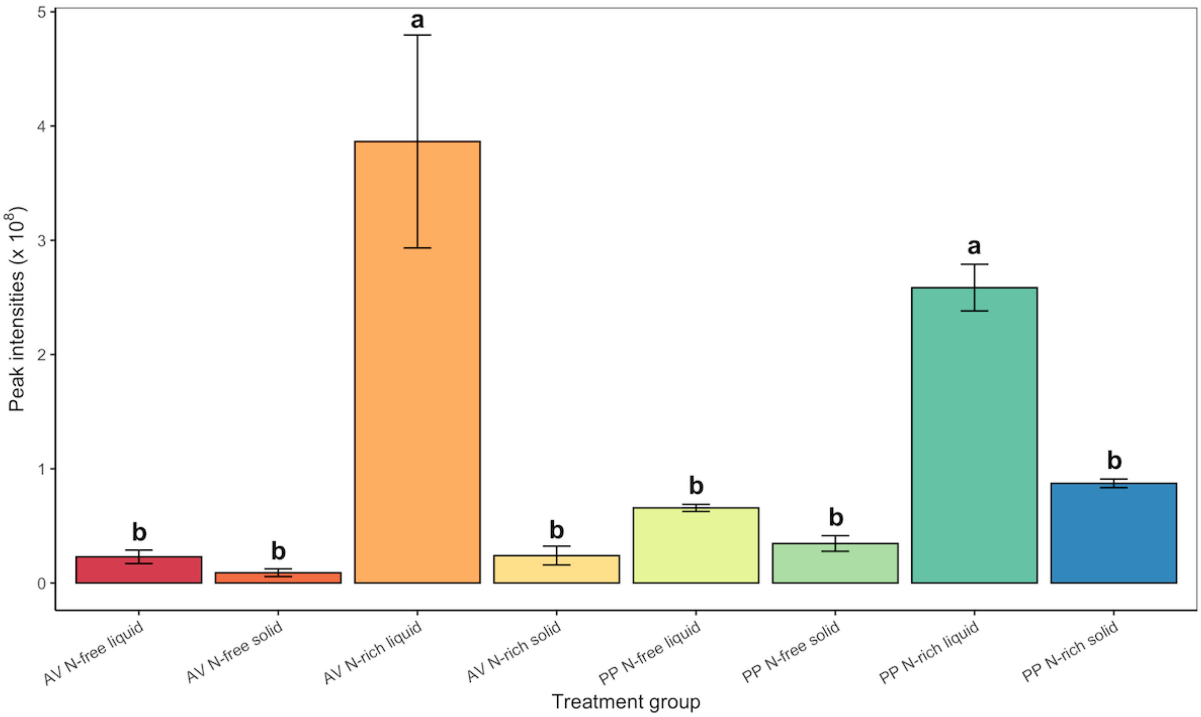

Fig. S3: Peak intensities of N-containing metabolites detected across treatment groups. Bars represent average peak intensity ± standard error. Lowercase letters indicate significant difference at $p < 0.05$.

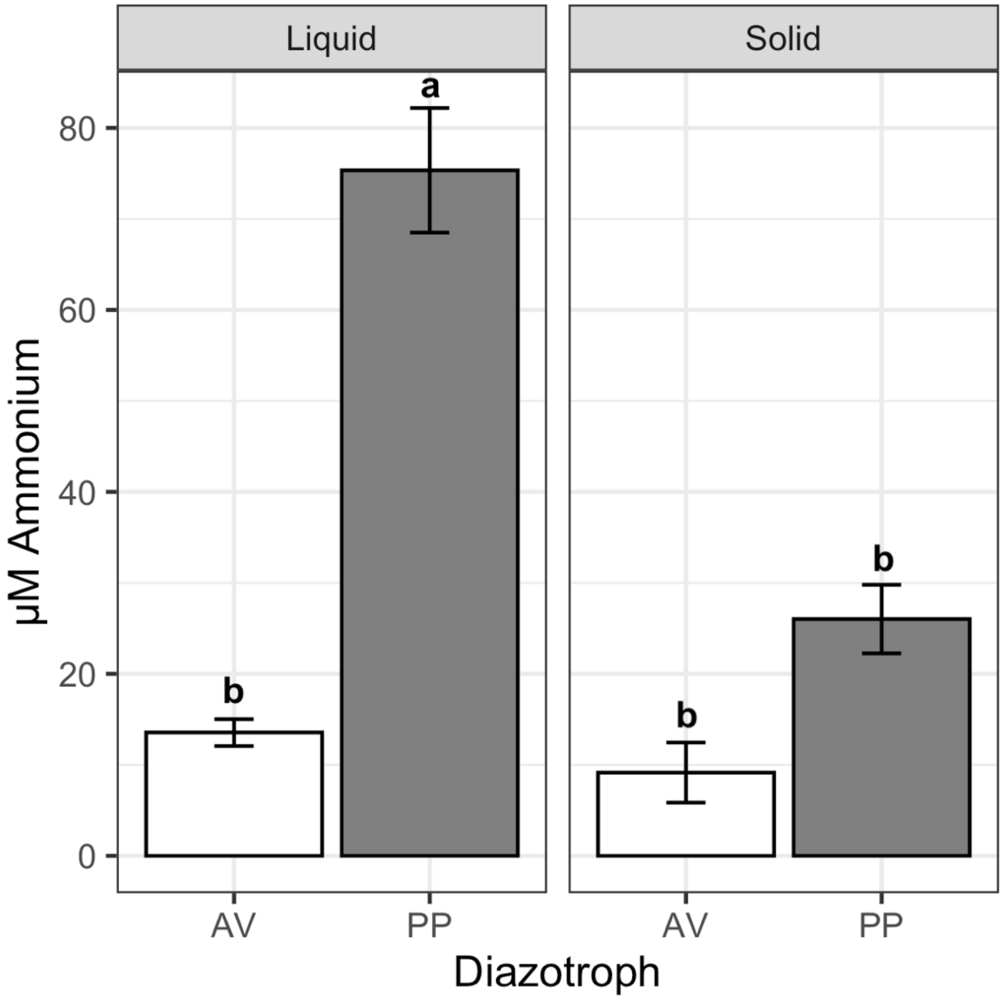

Fig. S4: Extracellular ammonium availability in µM for N-free treatments. Bars represent average values ± standard error. Lowercase letters indicate significant difference within culture type (liquid or solid) at $p < 0.05$.

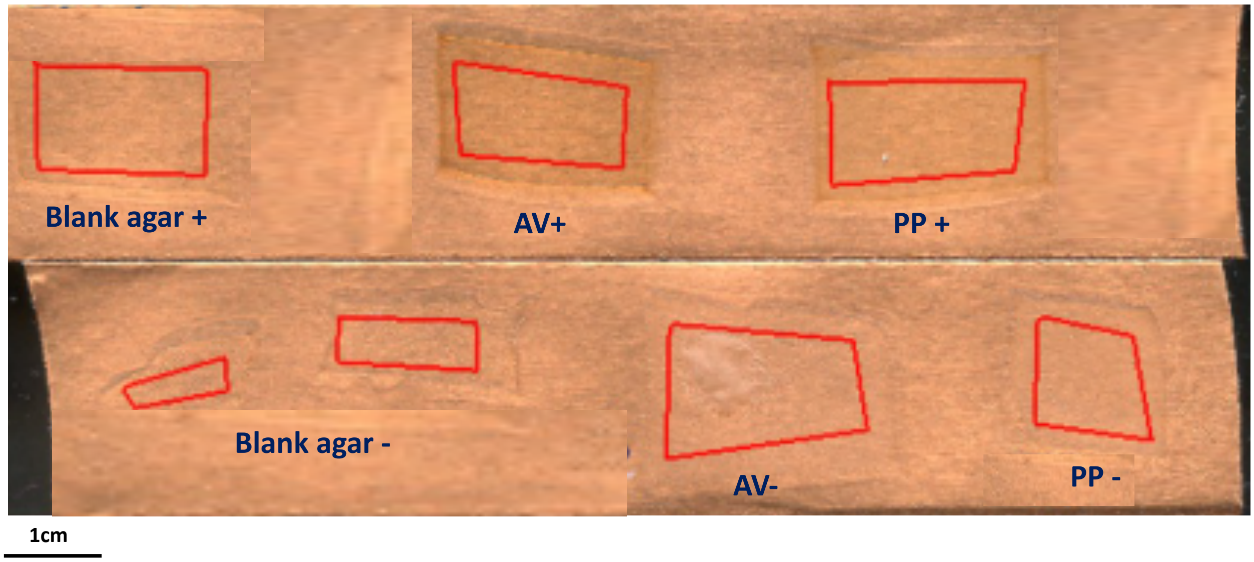

Fig. S5: Optical image of agar areas collected for MALDI MSI. Areas are placed on double-sided adhesive copper tape and adhered to indium tin oxide coated glass slides. Images were taken after regions were coated with MALDI matrix application. Red regions denote MALDI target areas within each sample.

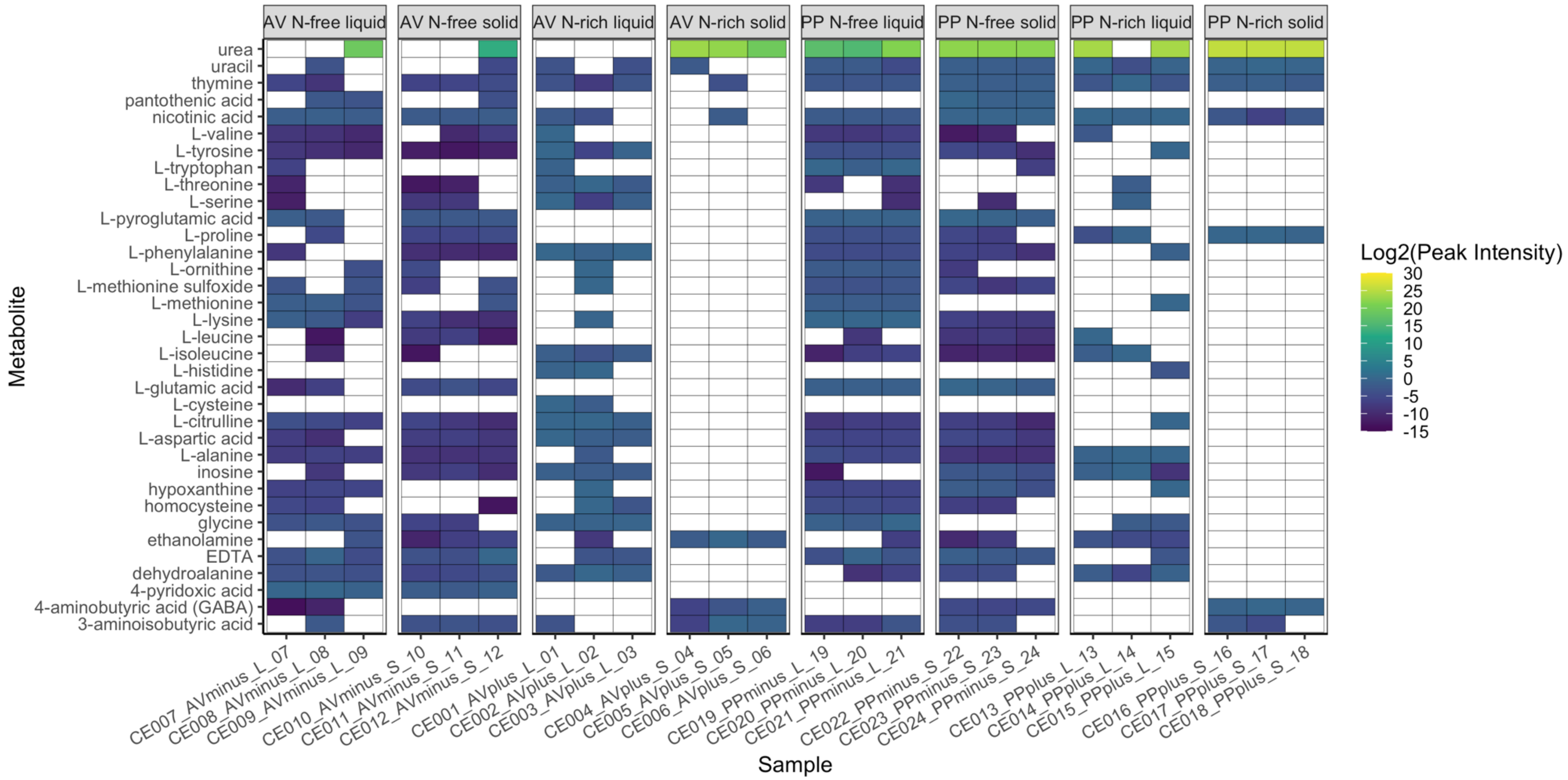

Fig. S6: Heatmap of N-containing metabolites and their peak intensities for all samples. Samples are grouped by treatment (organism [*A. vinelandii* (AV) or *P. polymyxa* (PP)], N treatment [N-free or N-rich], culture type [liquid or solid]). White cells indicate metabolite peak intensities was below detection. Peak intensity values are shown as log2 transformed to improve visualization.

Table S1: Area analyzed of each MALDI sample

Sample ID	Area analyzed (mm^2)	Raster size (µm x µm)	# of pixels spectra^{-1}
AV+	34.84	200 x 200	871
AV-	51.56	200 x 200	1289
PP+	43.60	200 x 200	1090
PP-	26.76	200 x 200	669
Blank+	42.48	200 x 200	1062
Blank1-	15.48	200 x 200	387
Blank2-	6.80	200 x 200	170
